# Detecting Fear-Memory-Related Genes from Neuronal scRNA-seq Data by Diverse Distributions and Bhattacharyya Distance

**DOI:** 10.3390/biom12081130

**Published:** 2022-08-17

**Authors:** Shaoqiang Zhang, Linjuan Xie, Yaxuan Cui, Benjamin R. Carone, Yong Chen

**Affiliations:** 1 Department of Computer Science, College of Computer and Information Engineering, Tianjin Normal University, Tianjin 300387, China; 2 Department of Biology and Biomedical Sciences, Rowan University, Glassboro, NJ 08028, USA

**Keywords:** scRNA-seq, differentially expressed gene, Bhattacharyya distance, memory formation

## Abstract

The detection of differentially expressed genes (DEGs) is one of most important computational challenges in the analysis of single-cell RNA sequencing (scRNA-seq) data. However, due to the high heterogeneity and dropout noise inherent in scRNAseq data, challenges in detecting DEGs exist when using a single distribution of gene expression levels, leaving much room to improve the precision and robustness of current DEG detection methods. Here, we propose the use of a new method, DEGman, which utilizes several possible diverse distributions in combination with Bhattacharyya distance. DEGman can automatically select the best-fitting distributions of gene expression levels, and then detect DEGs by permutation testing of Bhattacharyya distances of the selected distributions from two cell groups. Compared with several popular DEG analysis tools on both large-scale simulation data and real scRNA-seq data, DEGman shows an overall improvement in the balance of sensitivity and precision. We applied DEGman to scRNA-seq data of *TRAP; Ai14* mouse neurons to detect fear-memory-related genes that are significantly differentially expressed in neurons with and without fear memory. DEGman detected well-known fear-memory-related genes and many novel candidates. Interestingly, we found 25 DEGs in common in five neuron clusters that are functionally enriched for synaptic vesicles, indicating that the coupled dynamics of synaptic vesicles across in neurons plays a critical role in remote memory formation. The proposed method leverages the advantage of the use of diverse distributions in DEG analysis, exhibiting better performance in analyzing composite scRNA-seq datasets in real applications.

## 1. Introduction

Single-cell RNA sequencing (scRNA-seq) has been widely used as a tool to identify and characterize novel cell types and cellular mechanisms by profiling transcript abundance at the resolution of an individual cell. One of the important steps in understanding scRNA-seq data is the application of differential gene expression analysis. Many methods are currently employed to detect differentially expressed genes (DEGs) and related enrichment of biological processes, including SCDE [1], MAST [2], scDD [3], D3E [4], Monocle2 [5], SINCERA [6], DEsingle [7], SigEMD [8], EMDomics [9], edgeR [10], DESeq [11], glmmTMB [12], NEBULA [13], and singleCellHaystack [14]. Although DEGs and novel biological insights have been uncovered in scRNA-seq experiments using these established methods, there is much room to improve both the sensitivity and precision of current DEG analysis.

Existing analysis pipelines are often challenged by both high noise rate and heterogeneity in scRNA-seq data. Experimental noise in scRNA-seq data is frequently elevated due to low RNA capture efficiency, amplification failure during sequencing, and low sequencing depth. As a result, lower abundance transcripts in scRNA-seq experiments are difficult to capture, often resulting in ‘dropout’ noise. Heterogeneity also poses a significant challenge for scRNA-seq analysis because the process of gene silencing and activation in a single cell is a stochastic, ultimately resulting in differing gene expression values in similar and neighboring cells [15,16]. Finally, neighboring cells in the same brain tissue or tumor can exhibit a large degree of heterogeneity from cell to cell [17,18,19], which results from the presence of different cell types and cellular statuses. Because of these inherent complexities of scRNA-seq experiments, distributions are not completely or accurately modeled by a single distribution, manifesting in major challenges to differential gene expression analysis.

Existing DEG analysis algorithms are primarily classified as two groups, model-based and distance-based. The model-based methods are mostly based on fitting the Poisson model, the negative binomial (NB) model or the zero-inflated negative binomial (ZINB) model [20]. In particular, DEseq2 employs a gene-specific shrinkage estimation for the dispersions parameter to fit a NB model [21]. GlmmTMB is an R package for fitting generalized linear mixed models with NB [12]. NEBULA employs a negative binomial mixed-effects model (NBMM) which introduces subject-level random effects [13]. Compared with NB model, the ZINB model employs one additional parameter to define the probability of a count being zero or being distributed as an NB distribution. For example, in SCDE [1], the observed reads counts of genes are modeled as a mixture of dropout events by a Poisson distribution and amplification components by a NB distribution [22]. DEsingle utilizes a ZINB regression model to estimate the proportion of the real and dropout zeros in the gene expression data [7]. As opposed to model-based methods, there are an alternate stream of distance-based and nonparametric methods frequently employed. SigEMD [8] and EMDomics [9] identify DEGs by calculating the earth mover’s distance [23] between the frequency histograms of genes in two cell groups. Other variations on this theme include a new nonparametric method, singleCellHaystack [14], uses Kullback–Leibler divergence to find DEGs in a part of cells that are non-randomly positioned in a low-dimensional space.

Experimentally, scRNA-seq platforms fall into two main categories, plate-based and droplet-based, depending on their method of single cell isolation. Droplet-based methods make use of unique molecular identifier (UMI) counts (e.g., 10× Genomics) [24,25], while plate-based methods make use of reads counts to quantify gene expression (e.g., SMART-seq2 [26]). To deal with ‘dropouts’, the most popular method makes statistical models for scRNA-seq counts using a ‘zero-inflated’ distribution [27,28,29]. However several studies have found that zero-inflation was suppressed by UMI counts [30,31], i.e., zero-inflation was not necessary for UMI count data produced by the majority of droplet-based platforms [32,33,34]. This may explain why edgeR, which was based on a NB model and designed for bulk RNA-seq data, is superior to some tools designed specifically for single-cell data for detecting DEGs in some scRNA-seq benchmark datasets [35]. A recent comparative study of DEG tools reported that the non-parametric methods can capture multimodality in scRNA-seq datasets, performing better than the model-based methods designed for handling zero counts [36]. Conversely, model-based methods can model dropout events well, and thus perform better in terms of identifying true positives and false positives. By taking advantage of both the model-based and distance-based strategies, it is possible to simultaneously address the multimodality, heterogeneity, and sparsity of scRNA-seq data.

The memory formation is one of the most essential functions of mammal brains to maintain information and recall it at a future time [37,38,39]. Remote memory is defined as memory signals lasting more than days or even months. In the 1950s, Milner and her colleagues hypothesized that the hippocampus is primarily involved in consolidating and recalling recent episodic-like memories, while some cortical regions are mostly implicated in remote memory processing [40,41,42]. A critical biochemical feature of memory consolidation is the requirement for coordinated regulation of gene expression in memory-related neurons [43]. Thus, maintaining the long-term transcriptional stability of memory-related genes in brain cells (specifically named as engram cells) is a central mechanism for responding to environmental signals [44,45]. scRNA-seq has been used to study the enduring molecular dynamics required for encoding contextual memory within engram cells [46,47,48]. By applying scRNA-seq to *TRAP2; Ai14* mice brains, Chen et al. recently studied the transcriptional signature 16 days after fear conditioning and found that heterogeneous transcriptional programs specific for different neuronal and non-neuronal (e.g., astrocytes and microglia) cells are involved in remote memory retrieval [48]. They specifically employed the Mann–Whitney U-test for DEG analysis and detected many DEGs in neurons by comparing neurons with non-neurons and non-fear conditions. Although the results are interesting, it remains unclear how reliable the DEG analysis is, and more importantly, to what degree are the results of different DEG methods consistent.

In this manuscript we demonstrate the development and validation of a new R package, named DEGman, to detect DEGs between two groups of scRNA-seq data using Bhattacharyya distance and testing multiple distributions. The validations of DEGman on both simulated and real scRNA-seq data show that DEGman has superior performance of balanced sensitivity and precision compared to nine popular methods. We applied it to scRNA-seq data of *TRAP2:Ai14* mouse neurons that include multiple subtypes of neurons with and without fear memory. We identified 25 DEGs in common across five major neuron subtypes with or without fear memory. The enrichment of DEG genes related to synaptic vesicle (SV) highlights that the coordinated regulation of SV plays a critical role in remote memory formation.

## 2. Materials and Methods

### 2.1. Overview of DEGman Method

**Figure 1:**
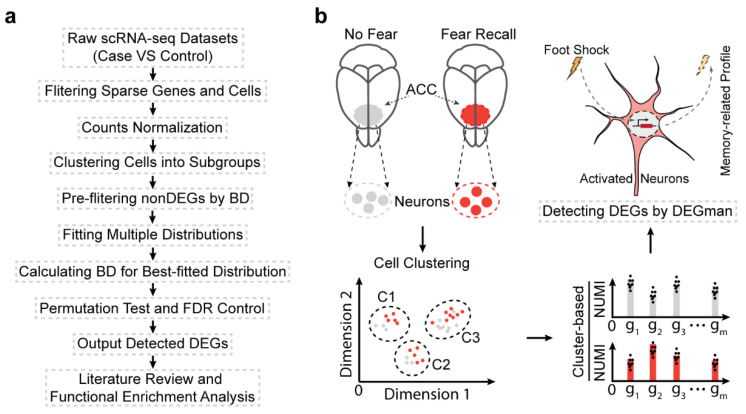
Overflows of DEGman method and detecting fear-memory-related genes. (**a**) Workflow of DEGman method. (**b**) The application of DEGman to detect memory-related genes from mouse neurons with fear memory. BD: Bhattacharyya distance NUMI: normalized UMI. ACC: anterior cingulate cortex.

To detect DEGs in scRNA-seq data with high heterogeneity and dropouts, a hybrid strategy was employed by optimizing the Bhattacharyya distance of three well-used distributions, NB, ZINB and Poisson (Figure 1a). First, genes without significant differences are quickly filtered by Bhattacharyya distance which is used to measure the similarity of two probability distributions. The Bhattacharyya distance has been widely used in research involving feature extraction and selection [49,50]. Second, for the retained genes, we fit expression levels with three distributions, NB, ZINB, and Poisson, respectively, to find the best-fitting distribution. We then compute the Bhattacharyya distance between the best-fitting distributions of the two conditions and do a permutation test.

The DEGman method was validated on large-scale simulated datasets and real scRNA-seq datasets. Specifically, it was applied to scRNA-seq data of 3530 *TRAP2:Ai14* mouse neurons to detect memory-related genes, i.e., DEGs, which are significantly differentially expressed in fear versus non-fear conditions (Figure 1b). Such analysis is a fundamental and critical endeavor to detect memory-related genes and stable expression patterns associated with remote memories since their formation and preservation depend on the coordinated regulation of memory-related genes in engram cells. By systematically investigating the memory associated DEGs, we aimed to identify the potential regulatory mechanisms underlying memory formation.

### 2.2. Data Preprocessing and Normalization

Given scRNA-seq datasets of two groups of cells, which include $m_{1}$ cells and $m_{2}$ cells, respectively, each element in row r and column c of its gene expression matrix is the UMI/reads count of a gene/transcript (row r) in a cell (column c). Preprocessing is performed to remove rows (genes) with all zero counts from the expression matrix and do log_2_(count + 1) transformation. Alternatively, the “NormalizeData” function in Seurat4 [51] with the “LogNormalize” method and a scale factor of $10^{4}$ or $10^{5}$ is also recommended to preprocess the input matrix.

### 2.3. Computing Bhattacharyya Distance

Given a gene with discrete probability distributions p(i) and q(i), i=0,1,2,…,N, in the two groups of cells, respectively, the Bhattacharyya coefficient [52] is a divergence-type measure between the two distributions, defined as

$$B\left(p,q\right)=\overset{N}{\underset{i=0}{\sum }}\sqrt{p\left(i\right)q\left(i\right)}$$

The following modification of the Bhattacharyya coefficient were proposed as a metric distance between distributions:

$$D\left(p,q\right)=\sqrt{1-B\left(p,q\right)}$$

Moreover, the value of D(p,q) was proven between 0 and 1 by Comaniciu et al. [53].

For a gene/row in the preprocessed matrix, N is the maximum integer by rounding off the row elements. p(i) and q(i) are the percentages of elements with rounding number i for two cell groups, respectively. 

In DEGman, we first computed the Bhattacharyya distance D(p,q) for each gene between two cell groups. It is clear that the Bhattacharyya distance is small if the expression distributions of the gene in two groups do not differ much. A distance threshold α was set for first round of filtering out gene rows that have only small difference between groups. In practice, α is set as 0.15. 

### 2.4. Best-Fit of Three Discrete Distributions

Chen et al. [31] analyzed a majority of mainstream scRNA-seq protocols and found that UMI counts can be modeled by simpler models (Poisson and NB) but ZINB model fits read counts better. Therefore, for each of the rest rows, we attempted to fit the genes in each group into the three discrete distributions, Poisson, NB, and ZINB, widely used in current scRNA-seq analysis. The functions “glm” and “glm.nb” from the R package MASS [54] were employed to fit Poisson and NB, respectively. The function “zeroinf” from the R package pscl [55] was used to fit the ZINB model. An NB model was fitted and then its parameters were used as the initial values of “zeroinf”. 

Chi-square goodness-of-fit test was employed to verify how close between the assumed distribution after fitting and the observed distribution [56]. The degree of freedom for the Chi-square test is n−p−1, where n is the number of discrete intervals and p is the number of parameters of the model used. That is, the degree of freedom for the ZINB model is n−4, that for the NB model is n−3, and that for the Poisson model is n−2. The assumed distribution with the highest *p*-value score of goodness-of-fit test was considered as the best fit of the observed data. The best fit was calculated for each gene row in each group. If a gene row did not converge to any of the three distributions, the empirical distribution of logarithmic read/UMI counts was used.

### 2.5. Permutation Test and FDR Control

For each gene row, the Bhattacharyya distance between the fitted distributions of two groups was calculated. The ZINB distribution is a mixture of constant zeros and a NB distribution with a mixture parameter [57]. In general, ZINB is not suitable for simulating gene expression levels that are generated in droplet-based scRNA-seq data [30], but there may be still a small proportion of genes whose best fit model is ZINB [31]. Furthermore, in DEsingle [7], the constant zeros in the ZINB model was shown to reflect the proportion of dropout zeros. Thus, we use the estimated parameters of the NB part to calculate the Bhattacharyya distance in droplet-based scRNA-seq data. If the distributions of gene expression levels failed to be fitted, the frequency distributions are used to calculate the Bhattacharyya distance.

To obtain the confidence of scores of Bhattacharyya distance, a permutation test to calculate the *p*-values was used. The null hypothesis is that there is no difference between the expression distributions of each gene in two cell groups. All cell columns were randomly shuffled into K permutations and divided into two groups of $m_{1}$ and $m_{2}$ cells in order, and for each permutation the Bhattacharyya distance between two cell groups for each gene is calculated. For a gene, given the distance score B between the original cell groups and K distance scores $\left(B_{1},\;B_{2},\;\ldots ,B_{K}\right)$ for K permutations, the *p*-value for the gene is computed as

$$p=\frac{\sum_{j=1}^{K}x_{j}}{K},\;\mathrm{where}\;x_{j}=\{\begin{array}{cc} 1, & \;\mathrm{if}\;B_{j}>B\\ 0, & \;\mathrm{elsewise} \end{array}.$$

To further filter out genes whose expression distribution was not significantly different under a default threshold, we used “p.adjust” function with the “fdr” parameter to adjust the *p*-values by FDR (false discovery rate) control [58]. The genes with adjusted *p*-values under 0.05 were selected as significantly DEGs. 

### 2.6. Datasets

Both simulated and real scRNA-seq datasets are used to evaluate the performance of DEGman and to compare it with other tools. DEGman was applied to the scRNA-seq datasets of *TRAP2; Ai14* mouse neurons with or without fear memory to systematically detect the fear-memory-related genes.

Simulated datasets: Since the true number of differentially expressed genes in real scRNA-seq experimental data cannot be known a priori, in order to assess the sensitivity and precision of the evaluated tools, we used a similar approach as Wang et al. [20] to generate a simulated dataset. First, the function “simulateSet” was used in the R package “scDD” [3] to generate simulated reads counts across two conditions each containing 75 single cells with 20,000 genes in each cell. Among the total 20,000 genes, 2000 genes were simulated as DEGs, which were equally divided into four groups, corresponding to the DU (differential unimodal), DP (differential proportion), DM (differential modality), and DB (both DM and DU) scenarios. The remaining 18,000 genes were simulated as non-differentially expressed genes, which were equally divided into two groups, corresponding to the EE (unimodal distribution) and EP (bimodal distribution) scenarios. To simulate the “dropout” events, for half of the genes in each scenario, we randomly implanted zero counts into each dataset according to a Binomial probability of 0.5 if the original data point was lower than the corresponding gene’s mean expression across all cell samples. Following this approach, we generated 10 simulated datasets.

Real scRNA-seq datasets: A real scRNA-seq dataset provided by Islam et al. [59] was used as the positive control dataset to compute true positive (TP) rates. It consists of 48 mouse embryonic stem cells (mESCs) and 44 mouse embryonic fibroblasts, whose count matrix is available in the Gene Expression Omnibus (GEO) with accession number GSE29087. We used the dataset created by Moliner et al. [60] through qRT-PCR experiments using the same cell types and culturing conditions. The validation dataset was downloaded from http://carlosibanezlab.se/Data/Moliner_CELfiles.zip (access on 10 February 2022) and was preprocessed using the Bioconductor package “affy” with Robust Multi-array Average (RMA) normalization. Only genes appearing as present in both data sets were used in this study. In several comparative studies of DEG-detection methodology [20,61,62], the top 1000 DEGs detected by the Bioconductor package “Limma” from the validation data were used as a gold standard gene set of the “positive control”.

A real scRNA-seq dataset provided by Grün et al. [63] was used as the “negative control” dataset to assess false positives (FPs). We retrieved 80 samples (cells) from mESCS identical condition (cultured in two-inhibitor (2i) medium) with 10,000 genes (GEO Accession No. GSE54695). The 80 samples were randomly shuffled 10 times to obtain 10 datasets. Each dataset was then divided equally into two groups, representing two conditions (e.g., case vs. control, 40 samples per condition). There should be no DEGs between any of the two groups in the 10 datasets due to the uniform cell type (all mESCS) under the same condition. 

ScRNA-seq datasets of neurons for memory study: scRNA-seq of neuronal cells of *TRAP2; Ai14* mice with or without fear memories was used to identify and study the transcripts of neurons involved in remote memory formation. 3530 Snap25+ neurons were collected after 16 days of fear conditioning vs. no conditioning and their transcriptomes were sequenced (GEO accession No. GSE152632) [48]. The Mann–Whitney U-test was used for DEG analysis and 94 DEGs were detected when comparing neurons with fear conditioning vs. no conditioning. Finally, the hierarchical clustering analysis of DEGs was performed using the Python package of scikit-learn 1.1.2.

### 2.7. Method Comparison

To benchmark DEGman, we considered eight popular tools, DEsingle [7], DEseq2 [21], SigEMD [8], scDD [3], edgeR [10], Monocle2 [5], glmmTMB [12] and NEBULA [13] which have been shown to have superior performance in multiple method comparisons [20,35,61,64,65]. We also compared DEGman with a new DEG finding method, singleCellHaystack [14], which uses the coordinates of all cells in a low-dimensional space produced by a dimensionality reduction methods, such as principal component analysis (PCA), t-distributed stochastic neighbor embedding (t-SNE), or uniform manifold approximation and projection(UMAP) [66,67]. According to the manual of singleCellHaystack, the top 50 dimensions (50D) of PCA, 2D of T-SNE, and 2D of UMAP were separately tested. The “nbiom2” family function was used in glmmTMB. NEBULA has two versions NEBULA-LN and NEBULA-HL. Here, we used NEBULA-HL because NEBULA-HL performed generally better than NEBULA-LN. Default parameters were set in DEGman, i.e., the threshold of Bhattacharyya distance was set as 0.15, the number of permutations K is 1000, and the threshold of adjusted *p*-values is 0.05. For all evaluated tools, the adjusted *p*-value is set as 0.05 and the other parameters were set as the defaults. Monocle2, DEsingle and Deseq2 directly used the reads/UMI counts as in input, the other tools used log-transformed read/UMI counts as the input. A summary of technical features, models and software versions of these methods can be found in Supplementary Table S1.

### 2.8. Criteria Used and Functional Enrichment Analysis

On both the simulated datasets and real datasets, we evaluate DEGman and other methods by calculating the true positive rate (TPR, or sensitivity), positive predictive value (PPV, or precision), true negative rate (TNR, or specificity), and F1-score that is the harmonic mean of precision and sensitivity. Here, the sensitivity is calculated as TPR=TP/(TP+FN), the precision is calculated as PPV=TP/(TP+FP), the specificity is as TNR=TN/(TN+FP) and the F1-score is calculated as F1=2*PPV*TPR/(PPV+TPR). The receiver operating characteristic (ROC) curve and the area under the curve (AUC) are calculated for these methods on the simulated data. For the DEGs detected from scRNA-seq of *TRAP2; Ai14* mouse neurons, we annotated DEGs functions by using Ensemble Biomart database [68]. Their functional enrichments were identified using David Database [69] and using GeneCards database [70].

## 3. Results

### 3.1. DEGman Has Superior Performance on Simulated Data

**Figure 2:**
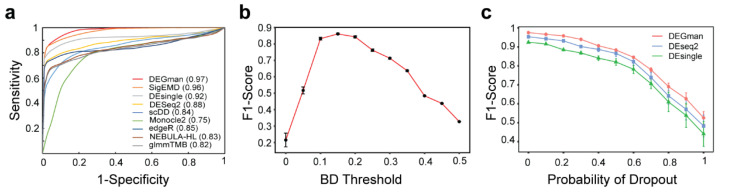
The performance of DEGman. (**a**) ROC curves and AUC values of nine DEG analysis tools using simulated data. (**b**) The F1-scores of DEGman on simluated data with different thresholds of Bhattacharyya distance. (**c**) The F1-scores of DEGman, DEseq2 and DEsingle for different dropout levels.

The DEGman package was evaluated on simulated data produced by scDD software (version 1.18.0, Bioconductor, Buffalo, NY, USA) widely used in studies for performance benchmarking. We compared DEGman with nine DEG detecting tools on the same simulated datasets using default parameters. For each of the 10 simulated datasets, the true DEGs recalled by a program from the 2000 DEGs were considered as TPs, the true DEGs not recalled by the program were considered as false negatives (FNs), and the predicted DEGs not from the 2000 DEGs were FPs. We plotted the ROC curves and calculated the AUC values of the nine tools. Results show that DEGman has a super AUC value of 0.97 compared with other tools (SigEMD: 0.96, DEsingle: 0.92, DESeq2: 0.88, edgeR: 0.85, scDD: 0.84, NEBULA-HL: 0.83, glmmTMB: 0.82, Monocle2: 0.75. Figure 2a). We evaluated the impact of DEGman on F1-scores at different thresholds of Bhattacharyya distance, and furthermore found that DEGman works best when the distance threshold is set between 0.1 and 0.2 (Figure 2b). The peak region of F1-scores demonstrated Bhattacharyya distance is a useful adjustment to filter non-signficant DEGs. 

Using a threshold of 0.05 adjusted *p*-value, we calculated the average numbers of predicted DEGs and TPs for each DEG analysis tool on the 10 simulated datasets. The average sensitivities, precisions, accuracies, and F1-scores of these tools were computed and listed in Table 1. Using these simulated datasets, DEGman has the highest F1-score of 0.861 among the ten tools, suggesting that it has a superior combined rate of TPs and FPs. Among these tools, Monocle2 can recall the most true DEGs, but it also contains the most FPs; DEsingle, DEseq2, and singleCellHaystack all demonstrate higher precision, but lower sensitivitiy than those of DEGman.

### 3.2. DEGman Exhibits High Sensitivity and Precision on Both Positive and Negative Control Experimental Data

All ten different DEG detection tools were run on the “positive control” real experimental dataset, containing 1000 genes as the gold standard DEGs. The numbers of detected DEGs and the true positives from the 1000 gold standard DEGs for each tool are listed in Table 2. DEGman captured 808 true positives, the most among the ten compared tools. Additionally, it was observed that, although the number of predicted DEGs of SigEMD, scDD, edgeR, NEBULA-HL, glmmTMB, and singleCellHaystack is less than DEGman’s, they also lost more TPs than DEGman.

Subsequently, all ten tools were run on the 10 “negative control” datasets. Hypothetically, for each “negative control” dataset, the optimal result for a DEG-detecting tool would be no identified DEGs. As shown in Table 2, most of these methods show extremely low FP rates, except of NEBULA-HL, glmmTMB, and Monocle2. DEGman reported only 5 FPs among 10,000 genes. Combining the results on positive and negative control real data, DEGman shows excellent performance in both TP rate and FP rate. For the other nine tools, some have either low FP rates, or low TP rates, or conversely, some have high TP rates with high FP rates.

### 3.3. Robustness against Dropouts and the Running Time of DEGman

To further verify the robustness of DEGman against dropout noise, the dropout noise levels was changed in simulation data by varying the probabilities p from 0 to 1 with a step of 0.1. Zero count data were randomly implanted into each data point according to a Binomial probability of p if the original data point was lower than the corresponding gene’s mean expression across all cell samples (Figure 2c). The results show that DEGman is very robust against dropout noise. For example, DEGman keeps an F1-score above 0.9 when the probability of dropout increases from 0 to 0.3. Moreover, it achieves a fair F1-score of 0.81 for a dropout probability of 0.6 which is considered a high level for most dropout levels of current scRNA-seq datasets. For further benchmarking, the robustness test was performed for DEseq2 and DEsingle methods that have F1-scores close to DEGman and better performances than other methods. The results show that DEGman has overall better robustness than DEseq2 and DEsingle (Figure 2c).

Since cell numbers are increasing to tens of thousands in some applications, we also evaluated the running times for these ten tools. We recorded the average running time on simulated datasets by a laptop with Intel Core i9 processors (Table 1). We observed that DEGman can output results within 567 s. Although singleCellHaystack is the fastest, it reported the lowest sensitivity and selectively recalled only genes with the strongest differential signals. Additionally, if the number of permutations of K of DEGman is reduced to 100, its accuracy is roughly unchanged, while the average running time can be reduced to around 60 s for the simulated data.

### 3.4. DEGman Identified Fear-Memory-Related Genes in Mouse Neurons

To identify the transcriptional profiles of neurons involved in remote memory, Chen et al. [48] sequenced *TRAP2; Ai14* mice expressing iCre-ERT2 recombinase in an activity-dependent manner along with a tdTomato (tdT) reporter allele, which can be used to label memory recall-activated neurons. The mice were exposed to tone-foot shocks on day 0 and induced fear memory recall (FR) on day 16. The control group was not fear conditioned but exposed to the recall context (no fear, NF). By comparing the gene expression levels of neuronal cells that were collected from FR and NF mice respectively, they identified 94 remote-memory associated DEGs (RA-DEGs). Here we reanalyzed this dataset by using DEGman and other nine DEG detecting tools whose performance are good on simulated datasets and real positive/negative datasets, to not only evaluate the consistency of results across these DEG analysis tools, but also detect new potential memory-related genes and mechanisms underlying remote memory formation.

**Figure 3:**
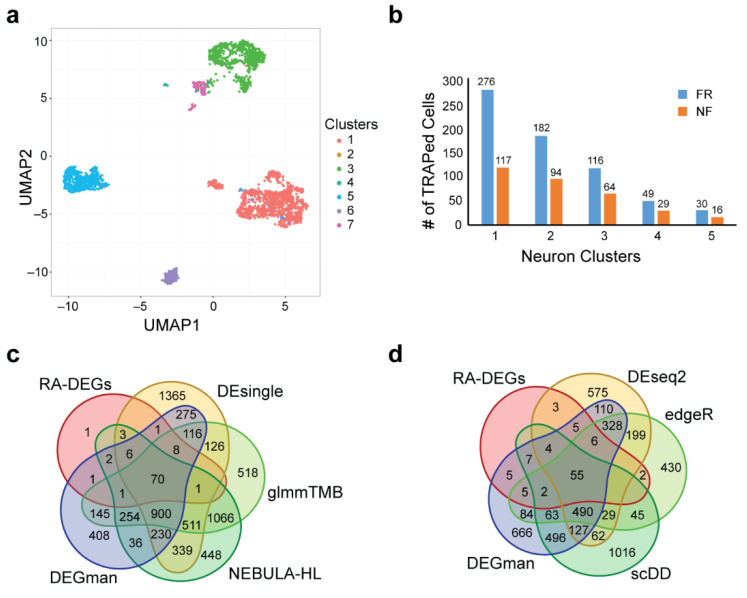
Comparative analysis of DEGs among mouse neurons. (**a**) UMAP dimensional reduction of the clustering result of all Snap25^+^ neurons (3530 cells). (**b**) The numbers of TRAPed FR and NF cells in the five largest clusters. (**c**) Venn diagram of the DEGs called by DEGman, DEsingle, glmmTMB and NEBULA-HL under an adjusted *p*-value of 0.05. (**d**) Venn diagram of the DEGs called by DEGman, DEseq2, scDD and edgeR under an adjusted *p*-value of 0.05.

First 3530 Snap25+ neurons were clustered by using the SCENA method [71] and the results were plotted via UMAP [67] (Figure 3a). The numbers of TRAPed FR and NF cells in the five largest clusters are shown in Figure 3b. For each of the five cell clusters, differential expression analysis was completed comparing TRAPed FR and NF cells using all eight methods. We united all detected DEGs together from the five clusters for each tool under an adjusted *p*-value of 0.05. The numbers of merged DEGs and the recalled RA-DEGs among them for each compared tool are listed in Table S2. In comparing across DEG tools, DEGman, Monocle2 recalled the highest number of RA-DEGs (90). Although Monocle2 recalled one more previous reported gene than DEGman and DEsingle, DEGman had the lowest number of predicted DEGs among the three tools (Table S2). The Venn diagram of DEGs predicted by the four tools of DEGman, DEsingle, NEBULA-HL and glmmTMB shows that DEGman also has the fewest uniquely predicted DEGs (Figure 3c), indicating that DEGman may have the lowest FP rate. Compared to DEGman, DEseq2, scDD, and edgeR predicted fewer DEGs, but missed more RA-DEGs. Additionally, we estimated that edgeR and DEseq2, which only fit the NB distribution, would reduce the FPs, but their TPs would also be decreased accordingly. The number of DEGs identified by DEGman was also compared to those of DEseq2, scDD, edgeR (Figure 3d) and we found that the RA-DEGs recalled by scDD were covered by DEGman, suggesting that DEGman should have the lower FP rate than scDD. Furthermore, DEGman overall identified more RA-DEGs than DEseq2 and edgeR. Notably, DEGman recalled more RA-DEGs while keeping and overall lower number of total predicted DEGs among the ten tools, achieving the highest Precision × Recall score (Table S2). These results further support that DEGman tends to show a better trade-off between sensitivity and precision than the other tools evaluated.

**Figure 4:**
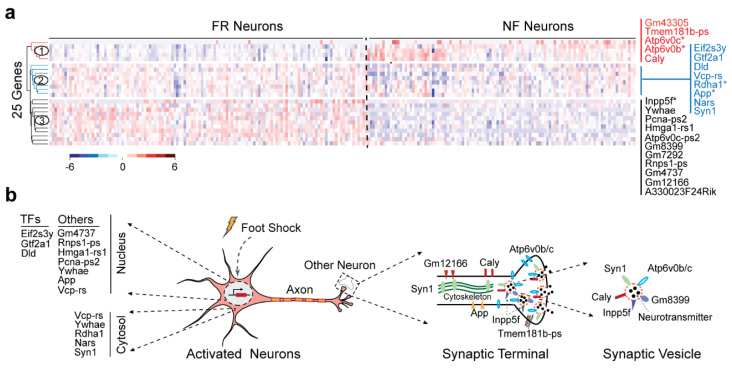
Expression heatmap and cellular locations of 25 fear-memory-related genes. (**a**) Heatmap of 25 genes detected by DEGman in the first cell cluster. Three gene groups are detected by hierarchical clustering analysis. * denotes the genes previously reported in RA-DEGs. (**b**) Illustration of gene locations. TF: transcriptional factor.

Among DEGs that are detected by DEGman from TRAPed FR and NF cells in the five largest clusters, there are 25 DEGs found in common including several ATPases, synapsins and several predicted genes (Table S3). The heatmap of the 25 DEGs showed a clear difference between TRAPed FR and NF cells (Figure 4a). Hierarchical clustering analysis shows that they can be divided into three gene clusters: cluster 1 shows lower expression levels in FR neurons and higher expression levels in NF neurons, while cluster 2 and 3 have higher expression levels in FR neurons and lower expression levels in NF neurons. Functional enrichment analysis indicates that they are enriched in the biological processes of mitochondrial acetyl-CoA biosynthetic process from pyruvate, hydrogen ion transmembrane transport, synapse organization and cerebral cortex development (Table S4). These results were also confirmed by KEGG pathway analysis as being enriched in pathways of the citrate cycle (tricarboxylic acid, TCA) and synaptic vesicle cycle. The enrichment of the mitochondrial acetyl-CoA biosynthetic process from pyruvate supports the long-term hypothesis that neurons themselves may de novo format transmitter glutamate from pyruvate carboxylation in vivo, leading to synthesis of TCA cycle intermediates, and thus keep the stable density of glutamate in neurons [72,73]. Although the activation-induced oxidative metabolism and glycolysis in neurons has been reported in early research [74,75,76,77], this is the first time that the regulation of such processes are observed from scRNA-seq data analysis.

### 3.5. Synaptic Vesicles Play Critical Role in Remote Memory Formation

Interestingly, the cellular component analysis of this scRNA-seq dataset indicates that these DEG genes are highly enriched at axons, within the nucleus, in the pyruvate dehydrogenase complex and in synaptic vesicles (SV). Taken together this may suggest that transmitter transport occurs at the synaptic connection terminals of axons when remote memory recalled (Figure 4b). SVs are small and electron-lucent vesicles that store neurotransmitters and release them by calcium-triggered exocytosis [78,79,80]. SVs have been shown to localize at the synaptic terminals and are regenerated after exocytosis. In particular, we found six genes, i.e., Syn1, Caly, Inpp5f, Atp6v0b, Atp6v0c, and Gm8399, annotated as SV-related genes by using GeneCards database [70] (Figure 4b). Syn1 is a member of the synapsin gene family that encodes neuronal phosphoproteins associated with the cytoplasmic surface of synaptic vesicles. The synapsins are implicated in synaptogenesis and the modulation of neurotransmitter release. Previous studies have linked synapins to the memory process and aging-related memory impairments in mammals [81,82,83]. Caly is known as a calcyon neuron specific vesicular protein that controls excitatory synaptic neurotransmission, vesical sorting, and synapse stability [84,85]. Inpp5f is predicted to be involved in protein transport and vesicle-mediated transport. Meanwhile, Atp6v0c and Atp6v0b are ATPases, involved in energy supply during SV cycle. Taking together, these results highlight that the dynamic regulation of genes are related to SV transportation and membrane trafficking during remote memory recalling. The analysis also demonstrates that the DEGman method was able to detect the biologically relevant DEGs and highlights the potential mechanistic discoveries underlying remote memory formation and recall.

## 4. Discussion

ScRNA-seq experiments have been widely applied to a range of broad biological questions, however, due to high dropout noise and heterogeneity, major challenges exist in DEG analysis of this type of data. To overcome these challenges, we propose a DEG detecting method, DEGman, which utilizes Bhattacharyya distance and testing multiple distributions. DEGman is shown to have superior performance compared to nine popular methods on both simulated and real scRNA-seq data. The high F1 score of DEGman demonstrates a better overall balance of sensitivity and specificity, and thus it is better able to identify true DEGs with important functions.

The benchmarking of ten tools on simulated data, real positive/negative control data and *TRAP2:Ai14* mouse brain cell data also provides a systematic assessment of the sensitively and precision of these tools. DEsingle, which is based on ZINB distribution performs well in simulated data but produces many FPs in real data. DEseq2 and edgeR which are based on negative binomial distribution have high precision, but relatively low sensitivity, so that some TPs are easily missed. NEBULA and glmmTMB were benchmarked to have outstanding performance for differential gene expression on simulated and real multi-subject scRNA-seq data of the 10x Genomics platform [64]. However, another reference on benchmarking methods for detecting differential states between conditions from multi-subject single-cell RNA-seq data shows the pseudo-bulk methods such as DEseq2 and edgeR performed generally best [65]. In this study, we found that, although NEBULA-HL and glmmTMB can achieve fair performance based on the simulated, real datasets and the *TRAP2; Ai14* mice brain cell data, their sensitivities, specificities, and F1-scores are not as good as DEGman, DEsingle, and DEseq2. Meanwhile, we observed that NEBULA-HL has better sensitivity, specificity, F1-score, and running speed than glmmTMB.

As a case study, we specifically applied DEGman to a scRNA-seq dataset comprised of *TRAP2:Ai14* mouse neurons that include multiple subtypes of neurons with and without fear memory. DEGman identified 25 DEGs in common across five major neuron subtypes with or without fear memory. The functional enrichment of these 25 DEGs shows that they are related to the citrate cycle and synaptic vesicle cycle, highlighting that the coordinated regulation of SV plays a critical role in remote memory formation. Our results provide novel candidates for experimental study considering how the coordinated regulation of memory-related genes is involved in the formation and preservation of remote memories within a specific population of neurons. Overall, the results of large-scale validations and utility in a real case study demonstrate that the DEGman method can overcome the noise and heterogeneity inherent in scRNA-seq to yield a more complete and accurate set of DEGs compared to previous methods.

## Figures and Tables

**Table 1:** Performance comparison of ten tools on the simulated data. Adjusted *p*-value < 0.05.

Tools	Average DEGs	Average TPs	Sensitivity	Precision	F1-Score	Time (s)
DEGman	1697.3	1591.1	0.796	0.937	0.861	567.59
DEsingle	1609.2	1514.3	0.757	0.941	0.839	1581.48
SigEMD	1458.6	1226.4	0.613	0.841	0.709	3006.17
DESeq2	1411.2	1335.8	0.668	0.947	0.783	88.77
scDD	1236.4	1092.6	0.546	0.884	0.675	3344.19
Monocle2	4883.5	1672.3	0.836	0.342	0.486	191.71
edgeR	1254.3	1163.2	0.582	0.927	0.715	25.41
singleCellHaystack	32	32	0.016	1.000	0.031	8.11
glmmTMB	1192.2	1045.6	0.523	0.877	0.655	1687.76
NEBULA-HL	1334.3	1194.2	0.597	0.895	0.714	94.34

**Table 2:** Numbers of predicted DEGs and the TPs of the 1000 gold standard genes for the ten tools, and numbers of detected DEGs (FPs) by using negative control real data. Adjusted *p*-value < 0.05.

Tools	TPs	DEGs	FPs of 10,000 Genes	FP Rate
DEGman	808	8175	5	0.0005
DEsingle	779	8242	4	0.0004
SigEMD	488	3702	51	0.0051
DEseq2	695	8437	19	0.0019
scDD	351	2638	5	0.0005
Monocle2	765	8674	917	0.0917
edgeR	580	4447	0	0
singleCellHaystack	238	1739	0	0
glmmTMB	417	3652	121	0.0121
NEBULA-HL	349	3947	114	0.0114

## Data Availability

Data used in this study are available in Gene Expression Omnibus (GEO) with the accession number GSE29087, GSE54695, GSE152632 (access on 1 February 2022). The R source code and instruction of DEGman are available at https://github.com/shaoqiangzhang/DEGman.

## Supplementary Materials

The following supporting information can be downloaded at: https://www.mdpi.com/article/10.3390/biom12081130/s1, Table S1: Compared software tools of DEG analysis; Table S2: The numbers of detected DEGs in 94 reported memory-related genes; Table S3: Detailed annotations of 25 DEGs; Table S4: Functional enriched categories of 25 DEG genes.
